# Imaging features and differential diagnosis of benign osseous cystic lesions: Aneurysmal bone cyst versus femoral neck herniation pit

**DOI:** 10.12669/pjms.42.7.15878

**Published:** 2026-07

**Authors:** Jisong Li, Huan Wu, Jianfei Du, Mengchen Liu, Yanjun Ma, Yun Wang

**Affiliations:** 1 Jisong Li Department of Ultrasound, Huzhou Shushan Geriatric Hospital, Huzhou, Zhejiang Province 313000, P.R. China; 2 Huan Wu Department of Pharmacy, Huzhou First People’s Hospital, Huzhou, Zhejiang Province 313000, P.R. China; 3 Jianfei Du Medical Department, Huzhou Shushan Geriatric Hospital, Huzhou, Zhejiang Province 313000, P.R. China; 4 Mengchen Liu Department of Radiology, Zhejiang Xinda Hospital, Huzhou, Zhejiang Province 313000, P.R. China; 5 Yanjun Ma Department of Tumor Rehabilitation, Huzhou Shushan Geriatric Hospital, Huzhou, Zhejiang Province 313000, P.R. China; 6 Yun Wang Department of Radiology, Huzhou Shushan Geriatric Hospital, Huzhou, Zhejiang Province 313000, P.R. China

**Keywords:** Aneurysmal Bone Cyst, Computed Tomography, Diagnosis, Femoral Neck, Herniation Pit, Imaging Features, X-Ray

## Abstract

**Objectives:**

To investigate the imaging characteristics and differential diagnosis of aneurysmal bone cyst (ABC) and femoral neck herniation pit (HP).

**Methodology:**

A retrospective analysis was performed of X-Ray and computed tomography (CT) imaging data from 28 patients with pathologically confirmed ABC and 31 patients with pathologically confirmed femoral neck HP, treated in Huzhou Shushan Geriatric Hospital between May 2022 and September 2025. The imaging characteristics of the patients were analyzed, and the efficacy of X-Ray and CT in distinguishing ABC from femoral neck HP was compared. The diagnostic efficacy of X-Ray and CT was assessed by Receiver Operating Characteristic (ROC) curves and the Area Under the ROC curve (AUC).

**Results:**

X-Ray was able to identify 24 ABC cases of cystic expansile bone destruction, two cases of pathological fractures, and uneven bone septa. Twenty-nine femoral neck HP cases presented as round/slightly round lucent areas with sclerotic margins in the femoral neck. On CT, all 28 ABC cases exhibited osteolytic bone destruction characterized by varying degrees of expansile changes and a broader spatial distribution within the femoral neck compared to HP. Of them, 26 had bone shells, and two had fluid-fluid levels. On CT scans, femoral neck HP lesions manifested as small, focal bone defects strictly confined to the anterolateral subcortical region of the femoral neck; these lesions exhibited homogeneous or fluid-like soft-tissue density, thin sclerotic margins, and a characteristic relationship with the cortex where focal slit-like defects were occasionally visible. X-Ray had an AUC of 0.778 (95% CI: 0.670~0.886), while CT had a higher AUC of 0.912 (95% CI: 0.839~0.986).

**Conclusion:**

There are distinct differences in X-Ray and CT imaging features between ABC and femoral neck HP. CT examination is more effective for the differential diagnosis of these two lesions.

## INTRODUCTION

Aneurysmal bone cyst (ABC) and femoral neck herniation pit (HP) are relatively common benign conditions that present as focal osteolytic hypodense lesions, with clinical symptoms ranging from mild to severe.^1^,^2^ Because of overlapping imaging phenotypes and the critical weight-bearing role of the proximal femur in surgical planning, accurate differentiation may not only guide decisions regarding biopsy, surgery, and treatment modality selection but also help prevent misdiagnosis of potentially malignant lesions as benign, thereby avoiding delayed diagnosis and treatment.^3^

ABC predominantly affects children and adolescents. Studies 2-4 indicated that primary ABC is closely associated with USP6-related genetic events, with typical imaging manifestations including expansile osteolytic destruction, thin shell-like cortical bone, internal septations, and fluid-fluid levels on MRI that are relatively common but lack absolute specificity. A retrospective study by Karaca et al.^5^ that included over two hundred cases further demonstrated that the imaging spectrum of ABC is influenced by factors such as lesion location and secondary nature, making reliable classification based on a single sign difficult. While pharmacotherapy for ABC, such as denosumab, is effective in some cases, treatment-related changes may impact correct diagnosis, further highlighting the importance of accurate initial imaging interpretation.^6^

Femoral neck HP, usually located in the anterosuperior subcortical region of the femoral neck, is mostly an incidental finding. As reported by Gao et al.,^7^ femoral neck HP typically appears on X-Ray and computed tomography (CT) as small, round or oval, hypodense foci with clear sclerotic margins. Some lesions exhibit tiny, channel-like cortical changes.^7^ Nuclear medicine studies have shown that femoral neck HP and related degenerative or stress-induced changes are important causes of asymptomatic focal increased uptake in the femoral neck.^8^ In addition, studies show that femoral neck HP is associated with morphological parameters of femoroacetabular impingement (FAI). Lee et al.^9^ reported that femoral neck HPs show a certain prevalence on hip MRI in asymptomatic populations and are correlated with the alpha angle. Arriaza et al.^10^ further confirmed a high detection rate of FAI-related imaging markers in professional athletes, providing evidence for the involvement of mechanical factors in the pathogenesis of femoral neck HP.

The differential diagnosis of ABCs is complex, as it can mimic or be secondary to other benign lesions, including femoral neck HP, as well as some malignant bone tumors, which can also present with a cyst-dominant appearance.^11^,^12^ Therefore, it is necessary to develop more practical differential diagnostic criteria based on conventional X-Ray and CT findings, incorporating features such as lesion location, marginal reactions, cortical continuity, and perilesional soft-tissue changes.

This single-center retrospective study systematically compared X-Ray and CT imaging features of ABC and HP and evaluated the value of these modalities in differential diagnosis. The study aimed to provide clearer evidence for standardized imaging assessment of proximal femoral cystic lesions and to reduce overtreatment or delayed treatment resulting from misdiagnosis.

## METHODOLOGY

In this retrospective analysis total of 66 patients with suspected cystic lesions of the femoral neck were initially screened from May 2022 to September 2025 conducted at Huzhou Shushan Geriatric Hospital. Following a rigorous selection process, five cases were excluded due to the lack of definitive pathological results, and two cases were excluded because of incomplete or poor-quality X-ray or CT imaging data. Ultimately, 59 patients (28 with primary ABC and 31 with femoral neck HP) were retrospectively enrolled. Given that femoral neck HP is typically an incidental finding, the specific indications for surgical curettage or biopsy in the HP group included persistent hip pain, relatively large lesion diameter, or atypical imaging features where tumor-like lesions could not be preoperatively excluded. All patients underwent both X-ray and CT examinations prior to intervention. (Ethical approval: 2025-026, Date: January 12^th^, 2026).

### Ethical Approval:

The study was approved by the institutional Ethics Committee on January 12, 2026.

### Inclusion criteria:

- Definite pathological confirmation provided by two senior bone pathologists using standardized histological criteria. ABC was identified by characteristic blood-filled cystic spaces separated by septa containing fibroblasts and multinucleated giant cells. Femoral neck HP was histologically defined as the herniation of fibrous connective tissue, synovial tissue, or fibrocartilage through a cortical defect into the underlying cancellous bone, typically accompanied by reactive trabecular sclerosis, according to the criteria established by Pitt et al.^13^
- Completion of X-Ray and CT examinations prior to surgery or core needle biopsy, with clear image quality sufficient for diagnostic analysis.
- For HP cases, co-existing femoroacetabular impingement-related deformities or degenerative changes were included as part of the typical clinical background.
- Complete clinical data.

### Exclusion criteria:

- Complicated with other bone tumors, infectious bone diseases, metabolic bone diseases, or secondary ABCs associated with primary lesions (such as giant cell tumor or chondroblastoma).
- History of prior fracture in the femoral neck region or treatments that may alter lesion morphology (such as surgery, radiotherapy, chemotherapy) before imaging examinations.
- Presence of image artifacts (such as motion artifacts, metal artifacts) that interfere with the accurate evaluation of lesion characteristics.

### Examination methods: X-Ray:

A Siemens AXIOM Aristos VX digital X-Ray machine was used, with projection positions adjusted according to lesion locations. For ABC patients, anteroposterior and lateral radiographs of the lesion site were obtained; for femoral neck HP patients, anteroposterior radiographs and frog-leg lateral radiographs of the hip joint were taken. The scanning parameters were set as follows: tube voltage 60–80 kV, tube current 50–100 mA, exposure time 0.02–0.1 s, image matrix 3072×3072, and pixel size 0.1 mm.

### CT examination:

A Philips Brilliance 64-slice spiral CT scanner was used, with the scanning range encompassing the entire lesion and surrounding normal tissues. The scanning parameters were as follows: tube voltage 120 kV, tube current using automatic milliampere technology (100–300 mA), slice thickness 1.0 mm, slice interval 1.0 mm, pitch 1.0, and scanning field of view (FOV) adjusted to 15–30 cm according to lesion size. After scanning, thin-slice reconstruction was performed with a 0.5 mm reconstruction thickness and a 0.5 mm reconstruction interval. Images were then transmitted to a workstation for multiplanar reconstruction (MPR) to obtain coronal, sagittal, and axial images for further analysis.

### Image analysis:

All X-ray and CT images were independently analyzed by two attending radiologists with over eight years of experience in musculoskeletal imaging diagnosis. To minimize potential bias, a rigorous blinding protocol was implemented: the radiologists were blinded to the pathological gold standard and all clinical data, including patient age, symptoms, medical history, and prior imaging reports. Although the anatomical location (femoral neck) was inherently visible on the images, the radiologists remained unaware of the specific pathological grouping during their assessment. The differential diagnosis was based on prespecified imaging criteria. For ABC, X-ray diagnostic clues included cystic expansile bone destruction and irregular bony septa, while CT criteria focused on osteolytic destruction with expansile changes, the presence of distinct periosteal bone shells, and characteristic fluid-fluid levels. For femoral neck HP, X-ray diagnosis relied on identifying round or quasi-round lucent areas with sclerotic margins; CT criteria emphasized subcortical bone defects strictly localized in the anterolateral region with homogeneous or fluid-like internal density and thin sclerotic margins. The recorded imaging features included lesion location, size (maximum diameter), morphology, margins, internal density, presence of septations, calcification, periosteal reaction, and perilesional soft tissue status. In case of diagnostic discrepancies, a consensus was reached through joint consultation and discussion.

### Diagnostic Efficacy Evaluation:

With pathological results as the gold standard, the accuracy, specificity, sensitivity, positive predictive value, and negative predictive value of X-Ray and CT in the differential diagnosis of ABC and femoral neck HP were calculated, respectively. Definitions:

### True positive (TP):

Pathologically confirmed as ABC and diagnosed as ABC by imaging examination;

### True negative (TN):

Pathologically confirmed as femoral neck HP and diagnosed as femoral neck HP by imaging examination;

### False positive (FP):

Pathologically confirmed as femoral neck HP but diagnosed as ABC by imaging examination;

### False negative (FN):

Pathologically confirmed as ABC but diagnosed as femoral neck HP by imaging examination.

Accuracy = (TP+TN)/(TP+TN+FP+FN) × 100%; Sensitivity = TP/(TP+FN) × 100%

Specificity = TN/(TN+FP) × 100%; Positive Predictive Value (PPV) = TP/(TP+FP) × 100%

Negative Predictive Value (NPV) = TN/

(TN+FN) × 100%

### Statistical analysis:

Data analysis was performed using SPSS 27.0 (IBM Corp., Armonk, NY, USA). The Shapiro-Wilk test was used to evaluate the normality of data distribution. Data with non-normal distributions were expressed as medians and interquartile ranges and analyzed using the Mann-Whitney U test. Categorical data were presented as numbers and percentages. Between-group comparisons of categorical variables were performed using Fisher’s exact test, whereas paired comparisons of the accuracy, sensitivity, and specificity of X-Ray and CT were performed using the continuity-corrected McNemar test.. Inter-observer agreement for qualitative imaging features (such as lesion location, margins, internal density, and presence of bone shells) was assessed using Cohen’s kappa coefficient (κ). The κ values were interpreted as follows: <0.20, poor; 0.21–0.40, fair; 0.41–0.60, moderate; 0.61–0.80, substantial; and 0.81–1.00, almost perfect agreement.

Receiver operating characteristic (ROC) curves were plotted, and the area under the ROC curve (AUC) was calculated to compare the diagnostic efficacy of X-Ray and CT. The comparison of AUCs between X-ray and CT was performed using the DeLong test to calculate the Z-statistic. P < 0.05 was considered statistically significant.

## RESULTS

This retrospective study included data from 59 patients (40 males and 19 females) who met the eligibility criteria. The age range of patients was 19–51 years, with a median age of 31 (25–36) years. There were 28 patients with ABC and 31 patients with femoral neck HP. Comparison of the general characteristics between the two groups showed no statistically significant difference (P>0.05) (Table-I). The two radiologists showed excellent consistency in identifying key imaging features. The Cohen’s kappa values for lesion location, margin characteristics, internal density, and the presence of bone shells were 0.93, 0.85, 0.82, and 0.88, respectively, indicating almost perfect agreement between the observers and ensuring the reproducibility of the diagnostic clues used in this study.

**Table-I:** Comparison of general characteristics between the two groups.

Characteristics	ABC (n=28)	femoral neck HP (n=31)	P
Sex, n(%)			0.403^a^
Male	17 (60.7)	23 (74.2)	
Female	11 (39.3)	8 (25.8)	
Age (years)	27 (22, 36)	34 (26, 38)	0.061^b^
BMI (kg/m²)	23.5 (21.7, 25.25)	23.2 (21.6, 25.1)	0.832^b^
Affected side			0.606^a^
Left	14 (50.0)	18 (58.1)	
Right	14 (50.0)	13 (41.9)	

***Note:*** a, Fisher exact test; b, Mann-Whitney U test.

**Fig.1:**
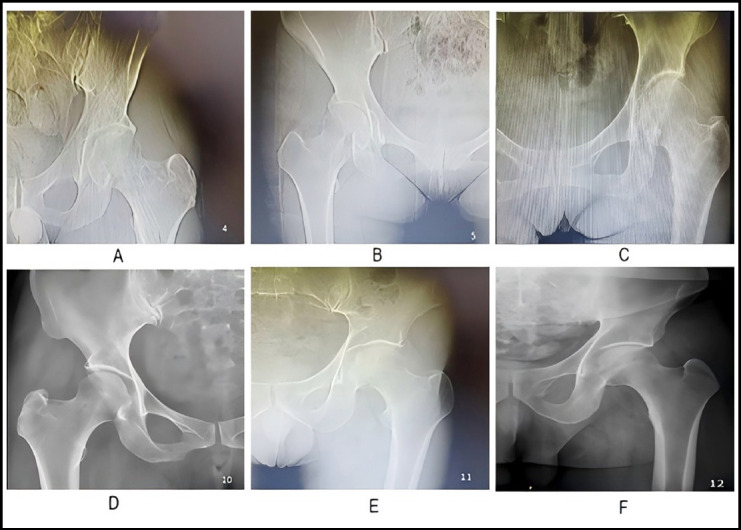
X-Ray findings of aneurysmal bone cyst and femoral neck herniation pit. (A-C) Pathologically confirmed ABCs, showing variable-sized osteolytic lesions of the femoral neck with heterogeneous internal density, partial internal septation, and variable cortical thinning or expansion. (D-F) Femoral neck HPs, showing small, round or oval subcortical radiolucencies with well-defined thin sclerotic margins and generally preserved adjacent cortex.

On X-Ray images, among the 28 ABC patients, 24 presented cystic bone destruction areas characterized by significant expansile growth that often spanned a larger diameter than HP, resulting in noticeable thinning of the adjacent bone cortex. Pathological fractures were complicated in two cases, with uneven bone septa visible inside. In contrast, among the 31 femoral neck HP patients, only 29 cases were detectable on plain X-Ray s, manifesting as smaller, round or quasi-round lucent areas typically confined to the subcortical region of the superolateral femoral neck, exhibiting a preserved cortical outline with thin sclerotic margins or rings and clear borders. Most lesions were situated in the femoral head base and the anterolateral subcortical region of the femoral neck (Fig.1).

**Fig.2:**
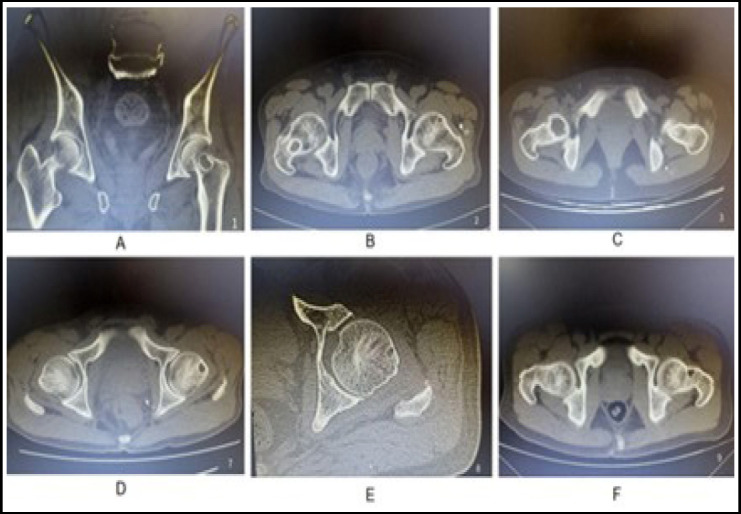
CT findings of aneurysmal bone cyst and femoral neck herniation pit. (A-C) ABCs showing expansile osteolytic destruction of the femoral neck, with cortical thinning or shell formation, heterogeneous internal density, and internal septation. (D-F) Femoral neck HPs showing small, well-circumscribed subcortical defects in the anterolateral femoral neck, with homogeneous or fluid-like internal density, thin sclerotic margins, and an occasional slit-like cortical defect.

On CT images, all ABC lesions presented as osteolytic bone destruction with varying degrees of expansile changes. Periosteal bone shells were observed in 26 cases, with heterogeneous internal density; fluid-fluid levels were detected in two cases. On CT scans, femoral neck HP lesions manifested as bone defects in the anterolateral subcortical area of the femoral neck, with homogeneous or fluid-like soft-tissue density within, clear boundaries, and thin sclerotic margins (Fig.2).

With pathological findings as the gold standard, the accuracy, sensitivity, specificity, PPV and NPV of CT in the differential diagnosis of ABC and femoral neck HP were 91.5%, 85.7%, 96.8%, 96.0% and 88.2%, respectively Table-II. All the above indicators of CT were higher than the corresponding values for X-Ray, which were 78.0%, 75.0%, 80.6%, 77.8%, and 78.1%, respectively. However, there were no statistically significant differences in accuracy, sensitivity or specificity between the two groups (all P>0.05). This lack of significance in discrete point estimates is likely attributed to the distribution of data at fixed diagnostic thresholds and the limited sample size.

**Table-II:** The efficacy indexes of X-Ray and CT in differential diagnosis of ABC and femoral neck HP.

Diagnosis	Accuracy (%)	Sensitivity (%)	Specificity (%)	PPV (%)	NPV (%)
X-Ray	78.0	75.0	80.6	77.8	78.1
CT	91.5	85.7	96.8	96.0	88.2
*χ^2^*	3.500	0.444	3.200	-	-
*P*	0.061	0.505	0.074	-	-

PPV: Positive predictive value; NPV: Negative predictive value.

**Fig.3:**
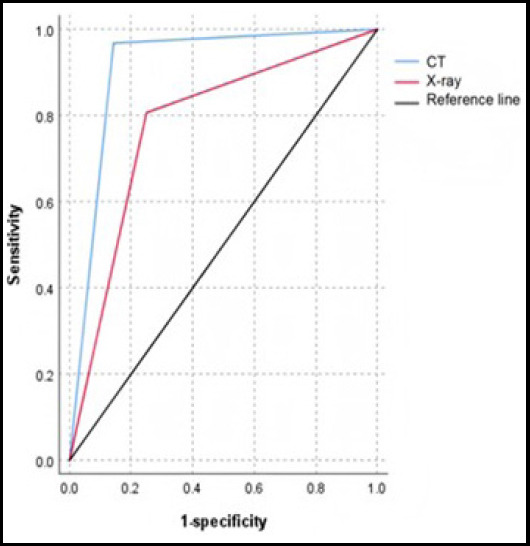
ROC curve.

ROC curve analysis showed that in differential diagnosis of ABC and femoral neck HP, the X-Ray and the CT examinations had an AUC of 0.778 (95% confidence interval (CI): 0.670-0.886), and 0.912 (95% CI: 0.839-0.986), respectively. There was a significant difference in AUC between the two groups (z = 2.123, P = 0.034), suggesting that CT examination is more effective than X-Ray examination for differential diagnosis (Table-III and Fig.3).

**Table-III:** The effectiveness of X-Ray and CT in the differential diagnosis of ABC and femoral neck HP.

Diagnosis	AUC	P	95%CI
X-Ray	0.778	<0.001	0.670~0.886
CT	0.912	<0.001	0.839~0.986

## DISCUSSION

This single-center retrospective study indicated that within the studied population, CT showed a trend toward higher effectiveness than X-Ray in the differential diagnosis of ABC and femoral neck HP. Although the point estimates for accuracy, sensitivity, and specificity were higher for CT, the differences did not reach statistical significance (all P > 0.05). However, CT had a significantly higher AUC than X-Ray (P = 0.034), indicating better discriminatory performance between ABC and femoral neck HP in this cohort.

The femoral neck is characterized by a delicate anatomical structure and significant overlapping projections, necessitating precise evaluation of cortical integrity and stability in imaging diagnosis.^14^,^15^

These results are consistent with previous research. CT, with its superior spatial resolution and structural visualization, better supports clinical decision-making regarding stratified follow-up, the need for additional MRI, or biopsy. Femoral neck HP is predominantly located in the subcortical region of the anterosuperior femoral neck, presenting as small round/oval defects with thin sclerotic margins and relatively homogeneous internal density.^13^ The findings of this study are consistent with the results of Gao et al.^7^ Previous reports have suggested an association of HP with FAI-related mechanical factors (such as increased alpha angle and repetitive impingement), leading to subcortical stress-induced changes.^9^,^10^ In nuclear medicine settings, focal increased uptake in the asymptomatic femoral neck is also frequently associated with hernia pits or degenerative changes.^8^ Therefore, when lesions exhibit typical location and continuous sclerotic margins, the ability of CT to identify the relation of subcortical defect and sclerotic margin with cortex can reduce the probability of misdiagnosing HPs as tumor-like lesions. Although this study focused on the overall diagnostic efficacy of imaging modalities rather than a standalone morphometric stratification, our findings emphasize that the combination of lesion location, maximum diameter, and cortical relationship remains the cornerstone of differential diagnosis. The predominantly subcortical, small-diameter nature of HP, coupled with its preserved (though occasionally cracked) cortex, contrasts sharply with the larger, more central or expansile nature of ABC which often compromises cortical integrity. CT’s superior performance, as demonstrated by our results, is largely due to its ability to precisely delineate these three key variables in the anatomically complex femoral neck region.

In terms of ABC diagnosis, the osteolytic destruction, expansile changes, and uneven internal structure with bony shells/septa observed in this study are consistent with the studies by Qadri et al.^4^ and Karaca et al.^5^ However, it is important to note that fluid-fluid levels lack specificity. A study by Van Dyck et al.^11^ confirmed their presence in various bone and soft tissue tumors. Furthermore, secondary ABC may overlap with features of the primary lesion, and chondroblastoma-associated secondary ABC carries a high risk of preoperative misdiagnosis.^16^ Additionally, denosumab therapy can alter the histological and imaging spectra of ABC/osteoblastoma, posing diagnostic challenges.^17^ The identification of novel USP6 fusion partners by Blackburn et al.^18^ further suggests that molecular re-evaluation should be considered when there is an imaging-pathological discrepancy or atypical biological behavior.

A cystic appearance does not equate to benignity. Telangiectatic osteosarcoma can be misdiagnosed as ABC preoperatively,^12^ and non-conventional subtypes of osteosarcoma can exhibit imaging phenotypes overlapping with those of benign cystic lesions.^19^,^20^ The imaging features of extraskeletal osteosarcoma may also be atypical due to necrosis/cystic change. A study by Wang et al.^21^ emphasized the need for a comprehensive assessment of soft tissue components and mineralization patterns. In this study, CT was more effective at identifying cortical breakthrough, soft-tissue components, or abnormal mineralization, thereby prompting further MRI or biopsy.

In complex or multifocal scenarios, such as multiple myeloma, low-dose whole-body CT, complemented by MRI, has been shown to improve assessment.^20^ Post-processing techniques, such as longitudinal bone subtraction proposed by Horger et al.,^22^ enhance the sensitivity of follow-up for subtle changes. In functional imaging, 18F-FDG PET/CT can detect soft-tissue ABC, which is associated with increased metabolism.^23^ Furthermore, differences among various PET tracers for bone metastasis detection highlight the need for cross-validation with morphological evidence.^24^

Current advances in imaging, combined with artificial intelligence (AI) and quantification, provide future directions for improving consistency. The advantages of machine learning are confirmed by the study by Guo et al.,^25^ which summarized the potential of deep learning for automatic triage in bone tumor screening. A study by Von Schacky et al.^26^ described a machine learning model based on X-Ray radiomics to assist in benign-malignant classification. Gersing et al.^27^ validated the feasibility of MR-generated CT-like images and simulated X-Ray, offering ideas for reducing radiation exposure and improving follow-up reproducibility.

### Strengths of this study:

It include using pathology as the gold standard, quantitatively comparing the diagnostic performance of X-Ray and CT, and summarizing operable CT differential points in the femoral neck, a region with significant imaging overlap.

### Limitations:

It includes single-center retrospective design and a relatively small sample size (n=59). The mandate for pathological confirmation as the reference standard inevitably introduced selection bias, favoring symptomatic patients or those with suspicious imaging findings that necessitated surgical intervention. Consequently, our cohort primarily represents clinically challenging cases rather than the incidental, asymptomatic HPs typical of routine practice. This bias is further reflected in the cohort’s median age of 31 years, which is notably higher than the typical adolescent demographic for ABC. Such a demographic shift is likely attributable to the adult-oriented referral patterns of our geriatric institution and the pathophysiological association of femoral neck HP with adult-prevalent conditions, such as femoroacetabular impingement. While this focus prioritizes the subpopulation where accurate differentiation is most critical for clinical decision-making, it may limit the generalizability of our findings to routine incidental cases or pediatric populations. Second, our analysis relied exclusively on X-ray and CT, without integrating systematic MRI sequences or quantitative parameters (such as DWI/ADC values and dynamic contrast-enhanced features). As a result, diagnostic signs that are more dependent on MRI—such as the sensitive detection of fluid-fluid levels and the evaluation of soft-tissue components—we’re not comprehensively addressed. Furthermore, the lack of external cohort validation and advanced modeling, such as radiomics or artificial intelligence, limits our ability to demonstrate the stability and reproducibility of the summarized imaging features across varying equipment parameters and institutional settings.

## CONCLUSION

This study demonstrated stable differences in imaging characteristics of ABC and the femoral neck HP on X-Ray and CT. Among these modalities, CT offers greater advantages in depicting the relationship between the lesion and cortical bone, marginal reactions, and internal structures. Within our samples, CT showed superior diagnostic performance compared to X-Ray in the differential diagnosis of ABC and the femoral neck HP, providing a preliminary imaging basis for the classification of benign cystic lesions of the femoral neck and supporting individualized clinical decision-making.

### Recommendations:

Given these inherent limitations, future large-scale, multicenter prospective studies are warranted to validate these preliminary findings and enhance the evidence level for broader clinical application.
